# Extensive dental caries in a HIV positive adult patient on ART; case report and literature review

**DOI:** 10.1186/s12903-018-0675-3

**Published:** 2018-12-07

**Authors:** Dunstan Kalanzi, Harriet Mayanja-Kizza, Damalie Nakanjako, Nelson K. Sewankambo

**Affiliations:** 1 Department of Dentistry, School of Health Sciences, Makerere University College of Health Sciences, P.O. Box 7072, Kampala, Uganda; 2 Department of Medicine, School of Medicine, Makerere University College of Health Sciences, Kampala, Uganda

**Keywords:** HIV, ART, Caries, Dentures, Cosmetic, Stigma, Psychological stress

## Abstract

**Background:**

The estimated number of people living with human immunodeficiency virus (HIV) (PLHIV) in Uganda is 1.5 million (7.3%). As of June 2016, 60% (898,197) of PLHIV were enrolled and receiving antiretroviral therapy (ART). In scientific literature, the effect of HIV and ART on dental caries remains equivocal. At the Prosthetics Clinic of the Department of Dentistry, Makerere University College of Health Sciences, we have seen a number of PLHIV who require replacement of missing teeth with partial or complete dentures as a result of extensive caries. Here we report a case of an HIV positive female patient with extensive dental caries resulting in complete edentulous jaws, associated with psychological stress and stigmatization.

**Case presentation:**

A 52-year-old patient, HIV positive for fourteen (14) years and receiving antiretroviral therapy (ART) for the last four years wanted to replace her missing teeth for effective feeding and cosmetic reasons. A diagnosis of partially edentulous maxillary and mandibular arches, cervical caries of tooth # 12, 15, 25, 34 and retained roots of tooth # 11, 13, 22 and 35 was made. Following oral health education and mouth preparation, this patient received a set of removable acrylic full upper and lower dentures.

**Conclusion:**

This case may represent the long-term effects of HIV and ART on oral health status especially tooth surfaces in some PLHIV. Further evaluation is required to ascertain if this was an isolated case or it is a common finding among HIV positive adult patients receiving long-term ART in sub-Saharan Africa. Information emerging from these studies would establish the magnitude of dental caries among PLHIV and guide the development of appropriate oral health care guidelines in the management of people living with HIV.

## Background

The HIV epidemic in Uganda continues to be generalized with the national HIV prevalence reported at 7.3%, which approximates to 1.5 million PLHIV. As of June 2016, about 60% (898,197) of HIV positive individuals were enrolled in HIV treatment programs and receiving antiretroviral therapy (ART), which is the gold standard in the treatment and prevention of HIV [1]. Until 2017 when the test and treat approach was adopted [2], the national guideline for ART initiation was a CD4 count of 250 cells/μl and below [3]. The use of ART has profound impact on the pattern of oral disease including a decreased prevalence of HIV-related oral lesions ranging from 10 to 50% [4, 5]. Dental caries is a dynamic pathological process that is primarily dependent on the development of virulent bio-films (plaque) formed on tooth surfaces from interactions of oral microbes (and their products), host salivary constituents, and dietary carbohydrates [6]. Saliva plays a significant role in oral and systemic health and its absence affects the quality of life. Individuals who suffer from salivary gland dysfunction are at risk of developing dental caries, periodontal diseases, and oral fungal infections [7]. Xerostomia and salivary gland hypofunction have been shown to be associated with HIV infection [8, 9]. This case report presents a unique clinical presentation of severe caries and missing teeth in a 52-year-old adult requiring replacement with complete dentures, for improved feeding and cosmetic reasons.

## Case presentation

A 52-year-old HIV positive patient presented to the Prosthetics Clinic of the Department of Dentistry at Makerere University College of Health Sciences in March 2017 with a desire to replace her missing teeth and remove the broken ones so that she could look aesthetically pleasing as well as improve her nutrition. She had been taking Tenofovir, Lamivudine and Efavirenz since 2014; and cotrimoxazole prophylaxis since 2004. She was generally in good health with no other chronic systemic illnesses. She reported having lost her first three teeth as a young girl resulting from tooth decay in the early 1980s’. Between that time and 2008, she lost two more teeth as a result of tooth decay. In 2008, she reported suffering from a severe febrile illness that left her bed ridden for two weeks, during which time she was unable to perform proper oral hygiene measures. Upon recovery, she noticed that her gums were bleeding and some of her teeth were loose and a number were lost (See Table 1 below).

**Table 1:** Self-reported loss history of between 1980 and 2017

Period	Teeth lost	Comments
1980s	Three (3)	Result of tooth decay
1980s-2008	Two (2)	Result of tooth decay
2003	–	Lost her husband and diagnosed with HIV
2004	–	Started on cotrimoxazole chemoprophylaxis
2008	Unknown but quite a number	As result of gum disease following severe febrile illness for two weeks
2014	–	Initiated on ART (Tenofovir disoproxil fumarate (TDF), Lamivudine (3TC) and Efavirenz (EFV)) at CD4 count ≤250 cells/μl, as recommended by the national ART guidelines at the time.
2015 -present	Rest of remaining teeth	Due to unprecedented tooth decay
2017	–	Rehabilitated with dentures (Fig. 3)

**Fig. 1:**
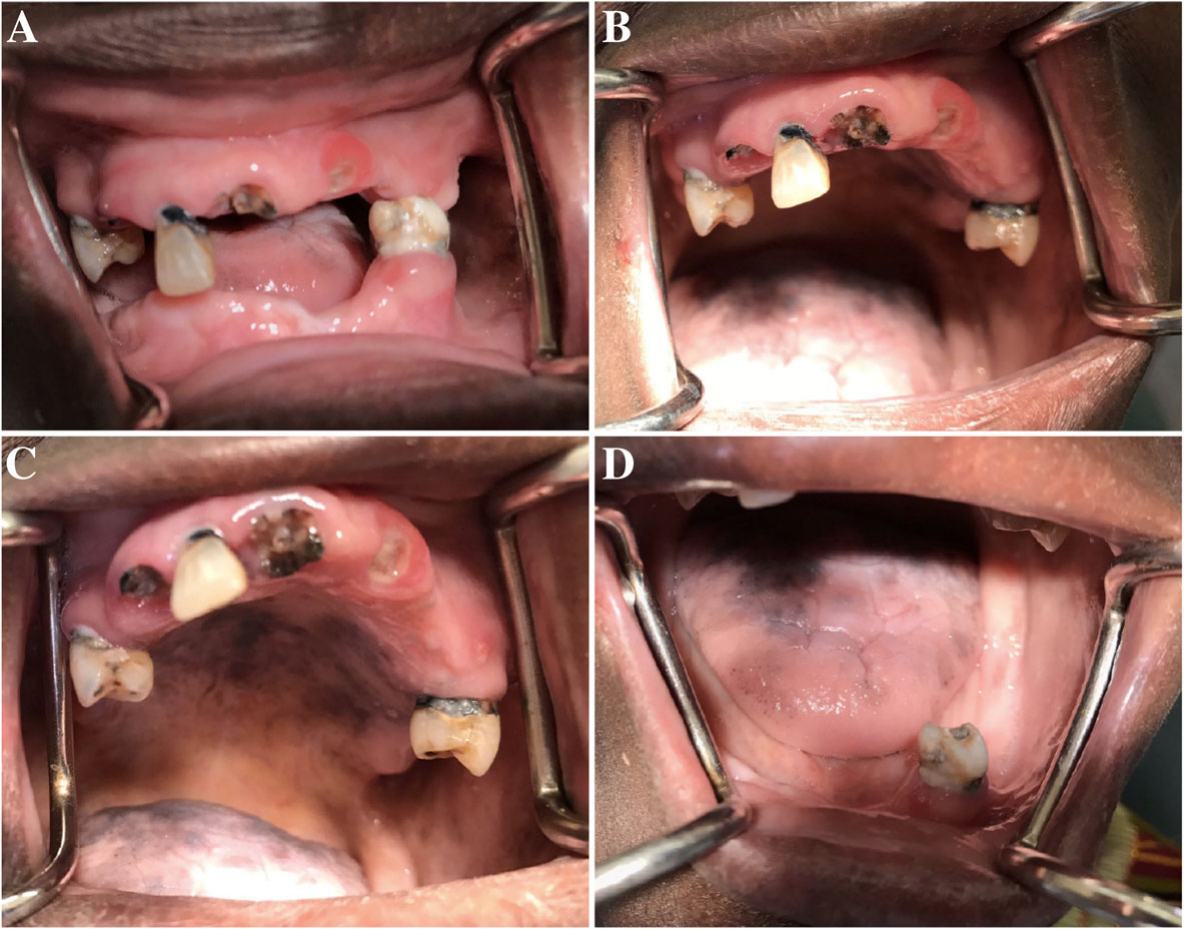
A and B, show inflamed upper labial marginal gingiva, cervical caries of the remaining teeth and retained roots secondary to caries. C and D, show diffuse blackish-purple pigmentation on the hard palatal surface and dorsum of the tongue probably secondary to ART or Kaposi’s sarcoma

**Fig. 2:**
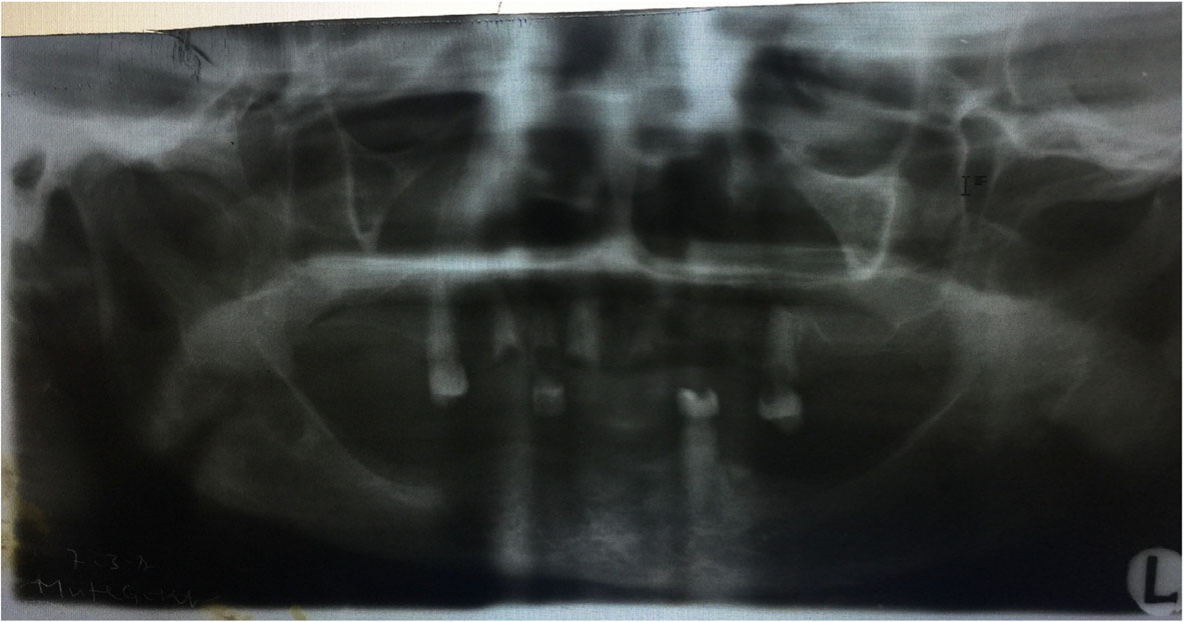
Orthopantogram showing the remaining teeth and retained roots

Between 2014, when ART was initiated until she presented to our clinic, she reported suffering from extensive tooth decay that caused more loss of teeth leaving her with just four teeth and four retained roots (Figs. 1 & 2). There is no history of smoking or alcohol consumption and a diet rich in refined sugars. Table 2 shows her CD4 and viral load measurements, as shown in her records at the HIV treatment centre.

**Table 2:** Annual CD4 count and Viral load measurements during ART, 2010–2018

CD4 monitoring		
Date	Cells or copies/ml	Comments
07/05/2010	505	Cotrimoxazole prophylaxis
15/11/2012	407	Cotrimoxazole prophylaxis
01/08/2013	378	Cotrimoxazole prophylaxis
23/08/2014	435	Started on ART (TDF/3TC/EFV)
VL monitoring		
17/12/2015	< 20	–
14/12/2016	Non detectable	–
16/03/2017	Non detectable	–
11/06/2018	Non detectable	–

Her diet consisted of predominantly high fiber carbohydrates including plantain, cassava, potatoes, rice, maize flour bread (posho) with fish, meat, beans, groundnut paste sauce and vegetables. As regards oral hygiene, she reported brushing twice a day using warm salt rinses.

On general examination, she was in fairly good general health condition without pallor of the mucous membranes, yellowing of the sclera or palpable cervical lymphadenopathy. The face was symmetrical with prominence of the zygomatic bones, sunken cheeks and mandibular prognathism, features associated with tooth loss. Temporomandibular joint (TMJ) examination showed no signs and symptoms of dysfunction.

Intraorally, there were light to moderate plaque deposits on the remaining dentition with diffuse blackish-purple pigmentation on the hard palatal surface and dorsum of the tongue probably secondary to ART or Kaposi’s sarcoma (Fig. 1). In the maxillary arch, tooth # 12, 15 and 25 were present with a peculiar type of cervical caries (caries affecting a significant portion of the cervix/neck of the tooth). There were also retained roots of tooth # 11, 13, and 22. In the mandibular arch, only tooth # 34 with the same peculiar cervical caries and retained root of tooth # 35 were present.

**Fig. 3:**
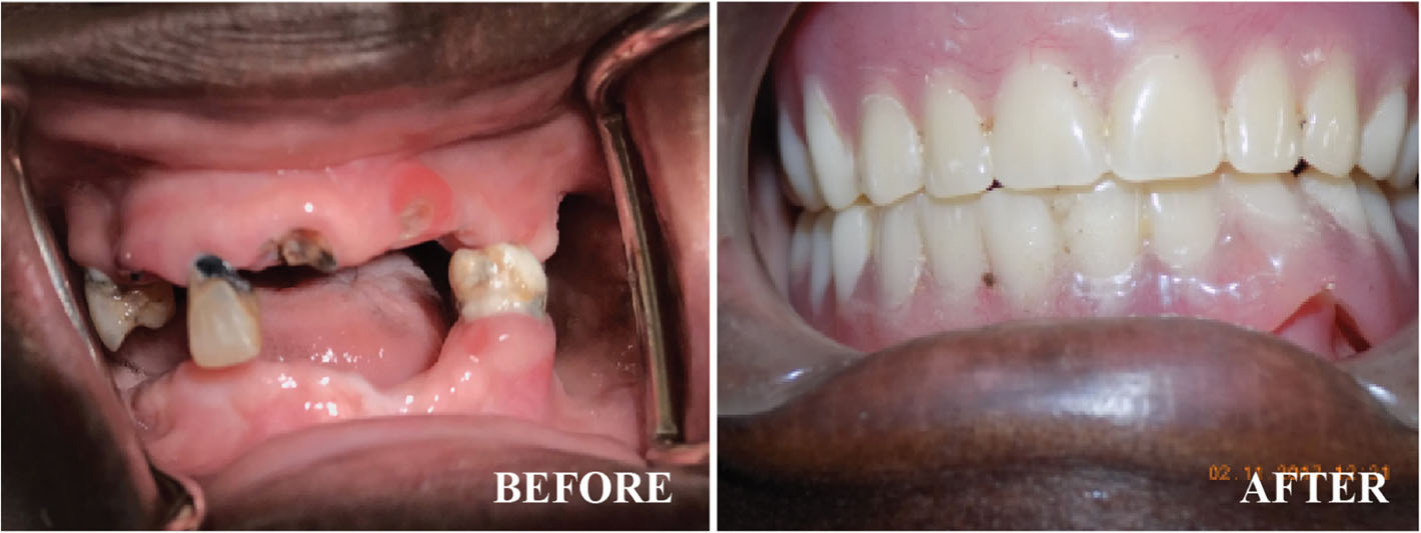
Before and after complete dentures to restore feeding and cosmesis

A diagnosis of partially edentulous maxillary and mandibular arches, retained roots of tooth # 11, 13, 22 and 35, as well as cervical caries of 12, 15, 25 and 34 was made. Following oral health education and mouth preparation, this patient received a set of removable acrylic full upper and lower dentures, which has significantly improved her feeding abilities and cosmetic concerns. (Fig. 3).

## Discussion

In this report we present the case of a female adult HIV positive patient receiving ART for two years and cotrimoxazole prophylaxis 13 years. She presented with severe dental caries and was rehabilitated with removable acrylic full upper and lower dentures. She desired to have her teeth replaced as a result of masticatory inability and social stigmatization due to her facial profile. It is well established that people with dentofacial abnormalities experience social consequences including greater degrees of social avoidance and being perceived as possessing negative personality characteristics [10]. Even minor facial abnormalities and hidden impairments such as total tooth loss can result in social stigma [11] and psychological stress comparable to visible facial disfigurements that have a profound effect on individuals [12], hence the need for replacement of missing teeth. The overarching need for replacement of the missing teeth was to improve aesthetics as a result of personal and societal pressure.

In this particular case, the patient reported that she suffered from a “severe caries” from the time she started her regimen of reverse transcriptase inhibitors although she had lost some teeth before that. In the literature, only two cases of severe caries associated with the use protease inhibitors and nucleoside reverse transcriptase inhibitors have been reported in HIV infected people [13, 14]. Dental caries is a dynamic pathological process that is primarily dependent on the development of virulent bio-films formed on tooth surfaces from interactions of oral microbes, host salivary constituents, and dietary carbohydrates [6, 15]. Documented studies from literature have shown that HIV infection is associated with xerostomia and salivary gland hypofunction [8, 9]. The infiltration of HIV and proliferation of CD8 lymphocytes in salivary glands along with the use of antiretroviral therapy (ART) decrease the salivary flow rate and change the normal microbial flora of the oral cavity [16]. It is also worth noting that this patient is on an ART regimen that includes tenofovir, which has been associated with loss of bone mineral density [17–20]. Whether what is seen in the long bones, joints and spine occurs in alveolar bone resulting in periodontal disease is an area that warrants research. Noteworthy is the fact that this patient’s dental caries dated back before ART initiation, hence the need for well-characterised studies to understand HIV-and ART-related and un-related risk of dental caries to inform targeted prevention and care interventions.

The limitation regarding this report is the quality of the orthopantogram (OPG). The x-ray machine accessed by our patients requires calibration for better results.

## Conclusions

This case demonstrates that people living with HIV may be susceptible to extensive caries that may be related to HIV and ART, and complete loss of teeth compounds the stigma associated with HIV/AIDS. Further studies are needed to investigate the mechanisms of increased risk of caries among PLHIV to inform development of appropriate oral health care guidelines.

## Abbreviations

3TC: Lamivudine
ART: Antiretroviral therapy
EFV: Efavirenz
HIV: Human immunodeficiency virus
OPG: Orthopantogram
PLHIV: People living with HIV
TDF: Tenofovir disoproxil fumarate
TMJ: Temporomandibular joint
